# Physics-Informed Masked Autoencoder for active sparse imaging

**DOI:** 10.1038/s41598-024-71095-x

**Published:** 2024-08-29

**Authors:** Luke McEvoy, Daniel Tafone, Yong Meng Sua, Yuping Huang

**Affiliations:** 1 Department of Physics, Stevens Institute of Technology, 1 Castle Point Terrace, Hoboken, NJ 07030 USA; 2 Center for Quantum Science and Engineering, Stevens Institute of Technology, 1 Castle Point Terrace, Hoboken, NJ 07030 USA; 3 Quantum Computing Inc., 5 Marine View Plaza, Hoboken, NJ 07030 USA

**Keywords:** Single photon detection, Sparse reconstruction, Artificial intelligence, Quantum optics, Engineering, Mathematics and computing, Optics and photonics, Physics, Applied physics

## Abstract

Imaging technology based on detecting individual photons has seen tremendous progress in recent years, with broad applications in autonomous driving, biomedical imaging, astronomical observation, and more. Comparing with conventional methods, however, it takes much longer time and relies on sparse and noisy photon-counting data to form an image. Here we introduce Physics-Informed Masked Autoencoder (PI-MAE) as a fast and efficient approach for data acquisition and image reconstruction through hardware implementation of the MAE (Masked Autoencoder). We examine its performance on a single-photon LiDAR system when trained on digitally masked MNIST data. Our results show that, with $1.8\times 10^{-6}$ or less detected photons per pulse and down to 9 detected photons per pixel, it achieves high-quality image reconstruction on unseen object classes with 90% physical masking. Our results highlight PI-MAE as a viable hardware accelerator for significantly improving the performance of single-photon imaging systems in photon-starving applications.

## Introduction

Images capture information representing the state of a physical scene. They are widely used to guide autonomous machines, inform medical diagnoses, survey land, and much more. For many machine vision applications, much of the captured information is redundant. For example, passively captured images often contain repetitive information and are therefore compressible, as exploited in several types of transform coding schemes such as the JPEG and MPEG standards. Additionally, in many cases most of the information contained in an image is irrelevant to its intended uses such as classification, segmentation, and movement tracking.

**Figure 1:**
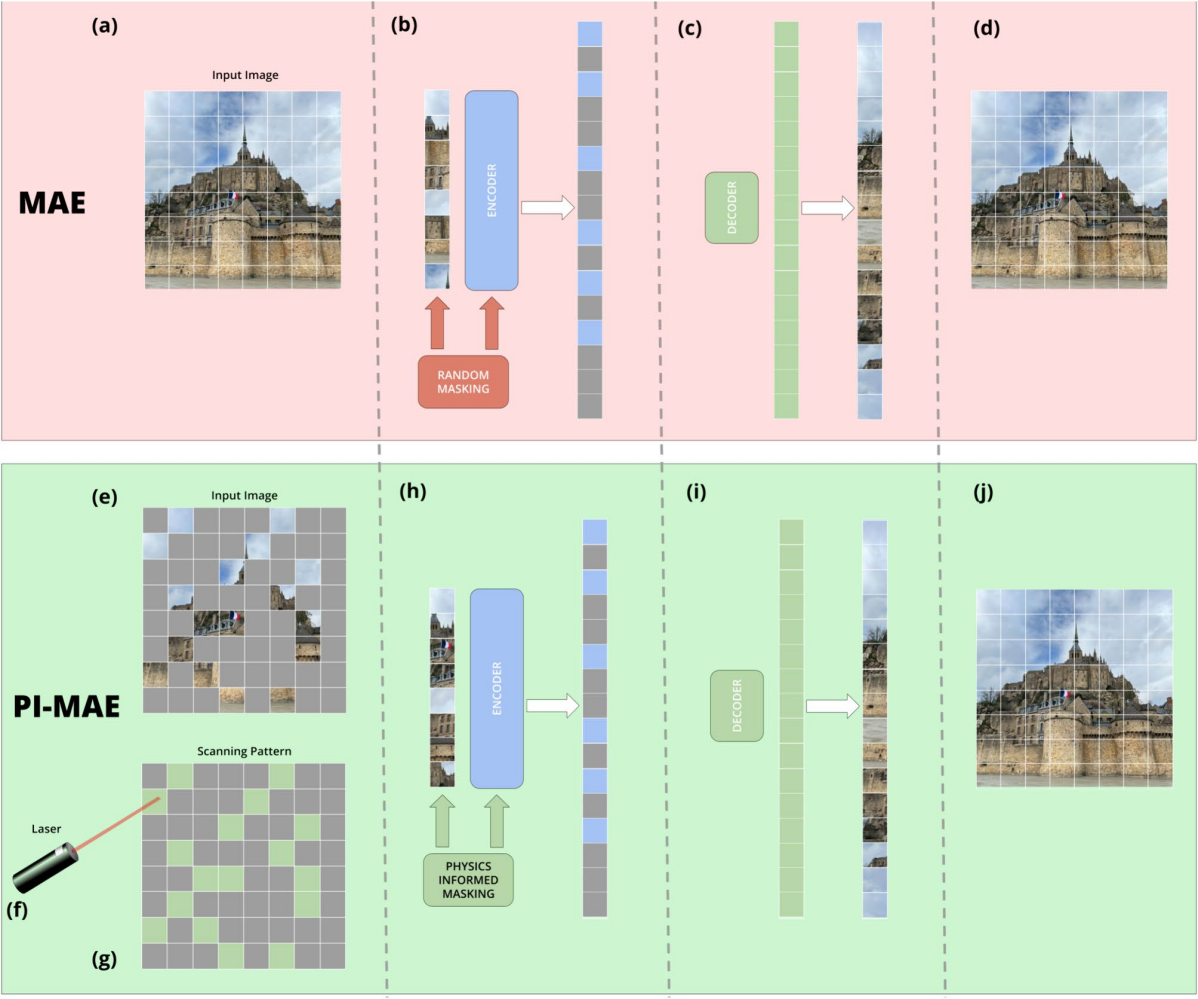
PI-MAE vs MAE architectures. PI-MAE uses the scanning pattern of laser to create physics informed masking in encoder. MAE randomly masks, with no information on where the laser scanned/what was masked. (**a**) Image input to the MAE model. (**b**) MAE encoder, which randomly masks the input image. (**c**) MAE decoder that reconstructs missing pixels. (**d**) Output image of the MAE model. (**e**) Input image into the PI-MAE model of an already masked (sparsely scanned) image. (**f**) Laser follows a scanning pattern. This scanning pattern is used by PI-MAE. (**g**) Input of the scanning pattern into the PI-MAE model for physics-informed masking. (**h**) PI-MAE encoder that employs physics-informed masking. (**i**) Decoder of PI-MAE that reconstructs missing pixels. (**j**) Output image of the PI-MAE model.

**Figure 2:**
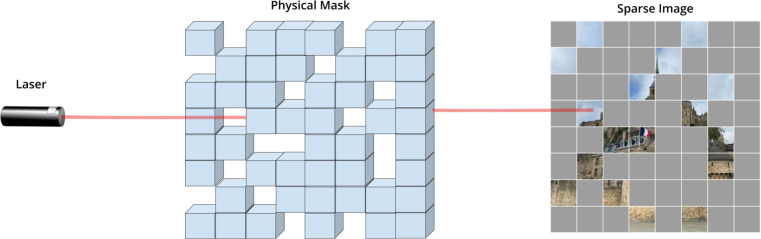
Sparse scanning of the single photon LiDAR for physical masking.

**Figure 3:**
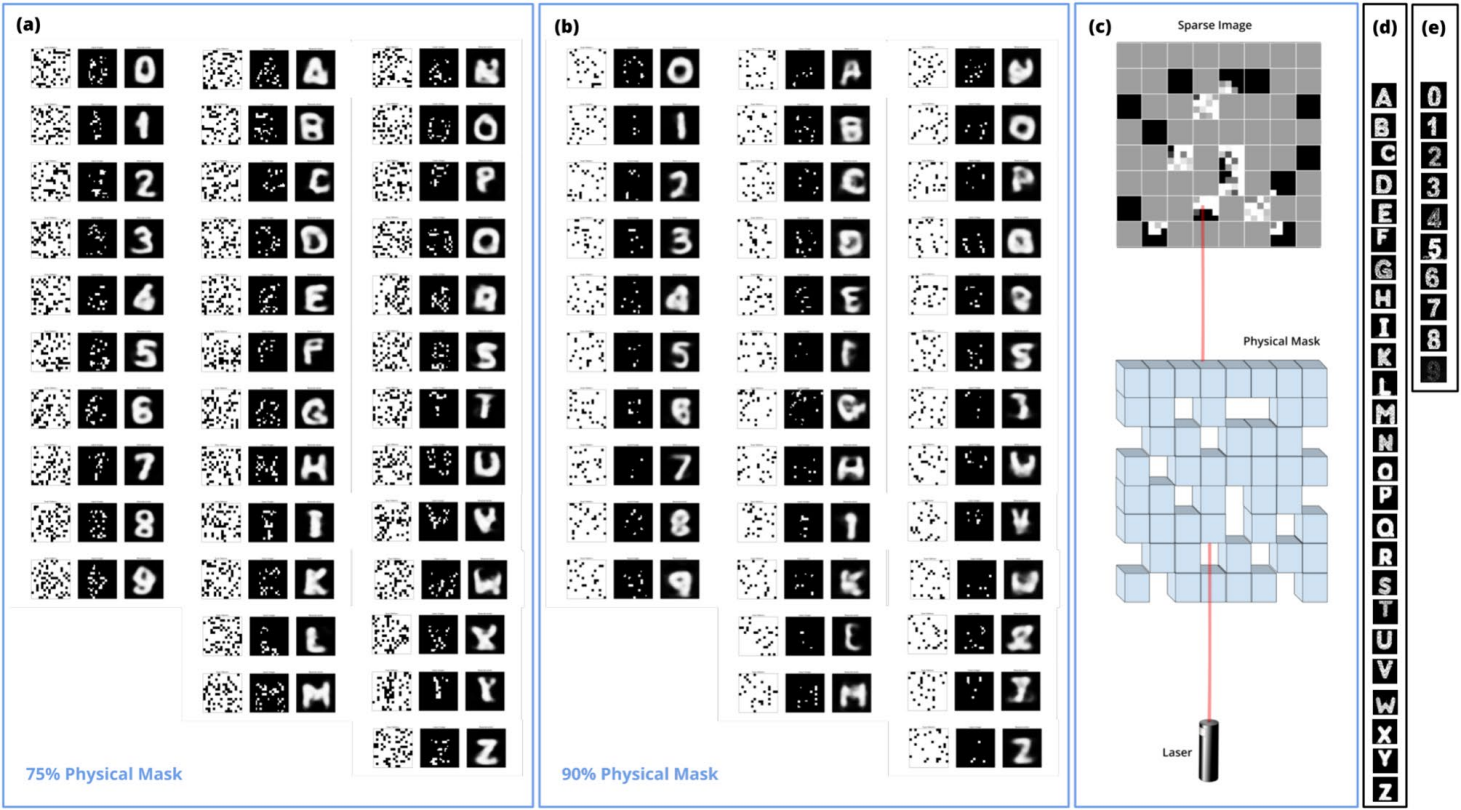
Reconstructed images by PI-MAE for (**a**) 75% physical masking and (**b**) 90% physical masking. In each group of (**a**) and (**b**), the leftmost column is the scanning pattern, the middle is the sparse image collected by the LiDAR system, the rightmost column is the reconstructed images. (**c**) An example acquired physically masked data. (**d**) and (**e**) are the ground-truth LiDAR scans for letters and numbers, respectively, which are what the data would have looked like if there had been no physical mask.

Such information inefficiency has inspired the development of systems which sample compressed representations of a scene. When applied to single-photon LiDAR, they promise to construct a whole image with photon counts from a small fraction of pixels, achieving high quality and frame rate despite rare and sparse signals. Also, extracting more information from less photons alleviates the demands on optical-to-digital signal processing speed and data storage volume. Thus far, compressive sensing systems have employed dynamic optical modulations using Spatial Light Modulators^1–4^, Digital Micromirror Devices^5,6^, coded apertures^7–9^, variational quantum sensors^10^, diffractive optical networks^11–15^, optical encoders^16^, and so on. Despite encouraging advancement, these modules are limited in speed, which restricts their use in fast-moving environments where the whole-scene information must be captured in a fraction of a second.

Here, we propose Physics-Informed Masked Autoencoder (PI-MAE), a hardware implementation of Masked Autoencoder (MAE)^17–19^. MAE is a self-supervised scalable computer vision model that *digitally masks* input images and reconstructs their masked pixels, as masked learning yields accelerated training and improved accuracy^17,20^. It has been widely deployed in reconstructing digitally masked video^21,22^, point cloud^23,24^, audio^25,26^, audiovisual^27^, and signal^28–30^. Motivated by MAE’s extensive successes and usefulness, PI-MAE applies the ideas behind MAE to compressive sensing by replacing the digital masking with *physical masking*. Rather than purposely omitting some pixels in already-taken images, PI-MAE fills in the missing pixels that are either physically obscured, or are not measured. In this sense, PI-MAE is to “unmask” the physically masked scenes, which implies two practical advantages. The first to provide information on physically inaccessible pixels and reconstruct the full images despite partially blocked views. The second is to reduce the amount of measuring pixels, thereby increasing the scanning speed in the case of LiDAR measurements. As such, a variety of PI-MAE applications are expected in sparse reconstruction of physically masked video, point cloud, audio, and signal processing. A comparison between PI-MAE and MAE is shown in Fig. 1.

We demonstrate PI-MAE in single-photon, single-pixel Light Detection and Ranging (LiDAR), an active imaging modality riddled with sparsity as it tappers with the fundamental particles of light: individual photons. It benefits significantly from ultrahigh detection sensitivity on a single-photon level and the ability to time-tag photon arrivals with nano-to-picosecond resolution. Recently, such technology and its derivatives have been deployed in remote sensing for millimeter to kilometer working distances^31–38^, with interesting applications in fluorescence Spectroscopy^39,40^, astronomy^41,42^, biomedical imaging of cancer and x-rays^43,44^, environmental imaging of photosynthesis and underwater scenes^45–47^, biometrics for reading heart beats^48,49^ and more. In this work, we down-sample an image plane via a random physical mask on the scanning pattern (i.e., scanning only a fraction of pixels) and reconstruct the full image by using PI-MAE that takes both the photon-counting results and the scanning pattern. In practice, such random masks can be chosen purposely—to speed up the scanning—or passively, because of line-of-sight obstructions, equipment impairments, and so on.

To examine its practical applicability, we first train PI-MAE on digitally masked numbers in MNIST, and later test it on unseen object classes of wood-cut letters and unseen data format: photon counts from LiDAR. Our results show that PI-MAE can successfully reconstruct those letters from LiDAR data. This is distinct to many deep learning-based approaches that cannot generalize well to out-of-distribution settings^50,51^. It also demonstrates that PI-MAE can learn the idea of shapes and a one-to-all mapping of sparse objects, and discover and exploit these hidden invariant features due to its higher representation power.

## Methods

### Physical system

We used a photon-counting LiDAR system to scan objects, specifically wooden numbers (0–9) and English alphabet. Unlike the MNIST dataset’s hand-drawn figures, our dataset comprised three-dimensional wooden blocks, featuring distinct fonts and dimensions, as shown in Supplementary Fig. 2.

The LiDAR system, illustrated in Supplementary Fig. 1, utilized a Mode-Locked Laser (MLL) emitting 6 picosecond pulses at 50 MHz repetition rate. The MLL provided electrical triggers to a gallium arsenide and indium arsenide (InGaAs) Geiger-mode Avalanche Photodiode (APD) single photon detector, enabling time-gated detection through synchronized electric pulses. A dense-wavelength-division-multiplexing (DWDM) system filtered these pulses to a singular infrared wavelength (1554.1 nm) for our experiments, although other wavelengths may be used. This wavelength, which serves as our signal, was then directed through a optic fiber circulator to a transceiver that emitted and received photons used for LiDAR scanning.

The emitted signal was a collimated beam of 2.2 mm diameter. It was reflected by a 45-degree mirror to a Micro-Electro-Mechanical Systems (MEMS) mirror, which then directed the beam onto the target object. The backscattered signal propagating along the same path is captured by the transceiver, passed through the circulator, and detected by the Single Photon Avalanche Detector (SPAD). This detector, set at a 5% efficiency, a 4.5 ns gate, a dark count rate of 2 kHz, and a 0.2 $\upmu$s deadtime, converted the photon counts into electrical signals. These signals were then processed by a Field Programmable Gate Array (FPGA), generating a Comma-Separated Values (CSV) file containing the photon counts from the FPGA.

### Data acquisition

In the experiment, each object was placed 1 meter away from the transceiver. Utilizing the MEMS mirror, we conducted linear raster scans over a 32 $\times$ 32 matrix, adjusting the beam’s angle by 0.28 degrees (equivalent to 2.2 mm on the target) per pixel to avoid overlap in the photon count data. Our integration time was 10 ms.

To execute physically masked LiDAR scans, we developed a Python script that randomly selects 2 $\times$ 2 patches within the 32 $\times$ 32 matrix for scanning, where the location of each patch yields a uniform random distribution. The selection percentage was set at 75% or 90% for our experiments, although the method can be adapted to any percentage. This patch-based scanning approach was implemented on the MEMS mirror, enabling automated scanning of targets.

### Data processing

Each csv file contained the transverse coordinates and registered photon counts for every pixel scanned. Any unscanned pixel was assigned to have zero count. Once the sparse scan is constructed to form an image, we binarize it by thresholding each pixel as follows. We first calculated the mean photon count ${\bar{N}}$ for each scanned pixel. Then, we chose the threshold photon count $\sigma$ as $\sigma = {\bar{N}} - \sqrt{{\bar{N}}}$, in observation of the shot-noise statistics of the photon counting. The grayscale value of each pixel was set to 0 if its photon count was less than $\sigma$, and set to 1 otherwise. This turns a photon-counting image into a binary image.

### Training protocol

PI-MAE was trained for 50 epochs on 1 NVIDIA T4 16GB GPU which took 45 min. The inference time was between 500 ms and 2 s. We randomly split MNIST (60,000 samples) into train (40,000 samples) and test (20,000 samples) sets. To directly apply the trained model to the experiment above, we divided each MNIST image into nonoverlapping patches of 2 $\times$ 2 pixels. During training, we randomly sampled a subset of patches, following a uniform probability distribution, while masking the rest. With this, PI-MAE learns how to reconstruct the missing pixels. Each reconstruction is compared to the ground truth image through a mean square error (MSE) loss function to guided self-supervised learning^17^.

### Network architecture

PI-MAE is an asymmetric autoencoder designed for the reconstruction of original signals from partially observed inputs, incorporating the scan pattern utilized during observation. This architecture comprises an encoder and a decoder, mapping observed signals to a latent space for subsequent reconstruction.

The PI-MAE encoder processes visible unmasked patches along with their corresponding scan patterns. It embeds these patches via linear projection, augmented with positional embeddings, and further refines the embedding through a series of Transformer blocks. In particular, the encoder operates on a reduced portion of the image plane, corresponding to the unmasked region (e.g., 10% for a 90% masking rate), thus improving efficiency by minimizing memory and computational demands.

The decoder receives a comprehensive token set, containing the encoded visible patches and mask tokens, which signify the locations of missing patches; see Sect. 4 in the Supplementary Materials. Positional embeddings are crucial here, providing spatial context to each token, including the mask tokens, within the image framework. The decoder employs transformer blocks to translate the latent representation back to the original input, for pixel-level reconstruction.

PI-MAE predicts the pixel values for each masked patch, and the decoder generates individual pixel values for each patch. A final linear projection layer adjusts the output to match the original image dimensions, facilitating the assembly of these patches into the reconstructed image. A more in-depth explanation of network architecture is in the Supplementary Materials 1, along with an illustration of self-attention in Supplementary Fig. 2, and a network diagram in Supplementary Fig. 3.

### Network hyper-parameters

The network was optimized by AdamW optimizer^52^ with a learning rate of $5\times 10^{-3}$ and a weight decay of $1\times 10^{-4}$. We also employ linear heating with cosine annealing, which is a learning rate scheduler^53^. The network batch size was 256, the buffer size was 1024, and the layer normalization was $1\times 10^{-6}$.

## Results

### Physically masked single photon imaging

After PI-MAE has been trained using masked MNIST data, we perform photon counting LiDAR measurement of both numbers and letters. As illustrated in Fig. 2, portions of the scene were physically blocked, by 75% or 90% in our experiments (which is the percentage of the plane that was never observed). The reconstruction results for 75% and 90% masking are shown in Fig. 3. We would like to note that PI-MAE has never seen letters before and no training was performed on real LiDAR data.

**Figure 4:**
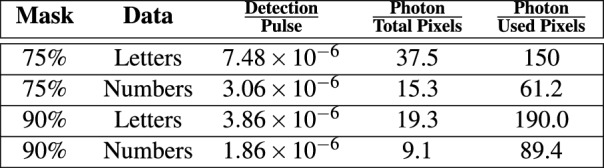
Physically masked single photon imaging statistics.

As seen, the characters were successfully reconstructed with a small number of photons captured in sparse pixels. In the case of 75% masking, the scenes were reconstructed with an average of 37.5 photons per pixel (i.e., total photon counts over total pixels include blocked ones) for the letters and 15.4 photons for the numbers. Likewise, for 90% masked scans of letters, we use 19.3 photons per pixel (blocked pixels included) and 9.1 photons per pixel for the numbers. We also computed detection per pulse, which is the number of photon counts multiplied by the integration time divided by the sampling frequency (50 MHz for our experiment). These results are summarized in Fig. 4.

In practice, noise photon counts can be registered over unscanned pixels. By physically informing the scanning pattern, PI-MAE ignores those counts so that they do not interfere with image reconstruction. To illustrate this, we added random photon numbers uniformly distributed between zero and the maximum registered count to the above 75% and 90% masked data. The reconstruction results are identical to Fig. 3, as shown in Supplementary Fig. 4.

**Figure 5:**
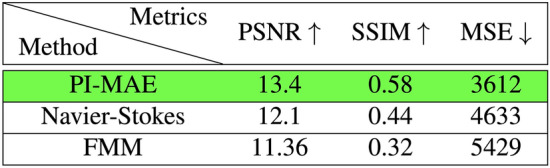
For 75% physical masking, PI-MAE quantitatively outperforms other methods inpainting methods: Navier–Stokes and Fast Marching Method (FMM), as shown by optimal PSNR, SSIM, and MSE scores.

**Figure 6:**
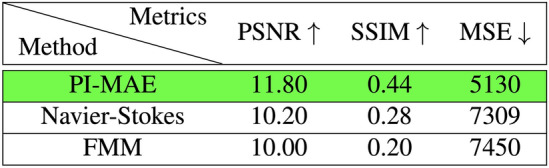
For 90% physical masking, PI-MAE quantitatively outperforms other inpainting methods: Navier–Stokes and Fast Marching Method (FMM), as shown by optimal PSNR, SSIM, and MSE scores.

Furthermore, we benchmarked PI-MAE against two industry standard algorithms. The first leverages the principles of fluid dynamics and the Navier-Stokes partial differential equations^54^. This method aims to probate edges from known to unknown regions, maintaining the continuity of edges and matching gradient vectors at the boundary. The second method utilizes the Fast Marching method to inpaint images^55^. The algorithm considers a small neighborhood around each boundary pixel, replacing the pixel with a normalized weighted sum of known neighboring pixels. The Fast Marching Method ensures that pixels near known regions are prioritized for inpainting. Next, we performed qualitative evaluations of PI-MAE’s reconstructions versus these two algorithms using three metrics: Peak Signal-to-Noise Ratio (PSNR), Structural Similarity Index Measure (SSIM), and Mean Squared Error (MSE), where PI-MAE outperformed rival metrics in all three categories. The numerical results are shown in Figs. 5 and 6. Furthermore, reconstruction results between Navier–Stokes, the Fast Marching Method and PIMAE can be seen for the 75% mask and the 90% mask in Supplementary Material Figs. 6 and 7 respectively.

## Discussion

We have demonstrated an agnostic image reconstruction architecture for sparse data, applicable to various imaging modalities. With about $10^{-6}$ or less detected photons per pulse and down to 9 photons per pixel, we demonstrated high-quality image reconstruction on unseen object classes with 90% physical masking. Our results highlight PI-MAE as a viable hardware accelerator for significantly improving the performance of single-photon imaging systems in photon-starving applications.

PI-MAE gives a number of practical advantages such as higher frame rate; fewer photons needed to be detected; less memory and energy consumption for data acquisition. It presents a solution to mitigate hardware limitations such as hot pixels, dark counts, and detector dead time, and provides a solution to imaging in photon-starved environments or physically obstructed scenes. It is an efficient compressive sensing method directly adoptable by LiDAR and other active imaging systems for medical, surveillance, astronomy, and environmental applications. In particular, we see PI-MAE flourishing in the autonomous driving industry, as the adoption of integrated photonic circuits of Optical Phased Arrays (OPAs) for LiDAR applications has spread widely over recent years^56,57^. The non-resonant, non-mechanical features of OPAs complement PI-MAE’s random physically down-sampled imaging methodology.

For future work, we will use a QPMS LiDAR system with a 36 dB signal-to-noise ratio improvement over the InGaAs single photon detector. This system leverages QPMS for noise rejection and a Si-APD for efficient photon detection, enabling active 3D imaging in high-noise environments^31^. Furthermore, we will use Time-of-Flight (ToF) histograms of photon detection to differentiate between masked and unmasked regions^38^.

Additionally, we expect diverse PI-MAE applications in sparse reconstruction of physically masked video, point cloud, audio, and signal processing in all domains. Furthermore, we expect a variety of PI-MAE adaptations utilizing information encoded in light’s spatial, spectral, or temporal degrees of freedom to perform sparse image reconstruction.

Although our training and testing datasets are relatively small and not complex, both masked autoencoders^17^ and vision transformers^58^ have shown that their performance scales impressively with increased data. This scalability is inherent to their design, suggesting that as PI-MAE is exposed to larger datasets, its ability to handle complex and diverse imaging tasks will similarly expand. This potential for scalability not only emphasizes the foundational strengths of PI-MAE in initial experiments but also suggests broad applicability in advanced imaging applications, from autonomous driving to medical imaging techniques.

### Novelty

To emphasize the novelty of PI-MAE, it is key to note MAE randomly masks patches without any reasoning or intelligence behind the selection. There is no coordination or communication between what is observed in the real world and which patches are masked in the digital world. In contrast, PI-MAE masks intelligently, with direct coordination and communication between real-world observations (where the laser beam is steered with the MEMS) and the digital world.

To illustrate this, consider a 32 $\times$ 32 image plane to be scanned. We divide this plane into 2 $\times$ 2 patches, resulting in 16 $\times$ 16 patches, or a total of 256 patches. If we skip/mask 90% of the patches, we are left with 26 patches to scan. If we scan these 26 patches and use MAE to reconstruct the image, the probability that the random digital masking aligns with the random physical masking is:

$$\begin{aligned} \frac{1}{\left( {\begin{array}{c}256\\ 26\end{array}}\right) } = \frac{1}{\frac{256!}{26!(256-26)!}} = \frac{1}{3.9 \times 10^{16}} = 2.6 \times 10^{-17} \end{aligned}$$

Each set of 26 patches is equally likely to be chosen, but there is only one specific set that matches what was physically masked by the MEMS/optical system. The probability that MAE chooses this set is extremely low, indicating that MAE will not align digital masking with physical masking.

However, with PI-MAE, there is no probability of masking overlap-it is guaranteed. The communication between the MEMS/optical system and the model ensures that physical masking always overlaps with digital masking, guaranteeing the highest fidelity in reconstructions and providing a more practical application. Ultimately, PI-MAE offers significant novelty and practicality over MAE.

### Supplementary Information

Supplementary Information.

## Data Availability

PI-MAE code and data is available for download at https://github.com/luke-mcevoy/PI-MAE.
