# Supplementary material for: Physics-Informed Masked Autoencoder for active sparse imaging
Source: Sci Rep. 2024 Aug 29;14:20078. doi: 10.1038/s41598-024-71095-x (PMC11362164; doi:10.1038/s41598-024-71095-x)
Supplement: Supplementary file 1 — Supplementary Information. [file 41598_2024_71095_MOESM1_ESM.pdf]

PI-MAE Supplementary

Luke McEvoy[1*], Daniel Tafone[1, 2], Yong Meng Sua[1, 2, 3], Yuping Huang[1, 2, 3]

[1*]Physics Department, Stevens Institute of Technology, 1 Castle Point, Hoboken, 07030, NJ, USA.
[2*]Center for Quantum Science and Engineering, Stevens Institute of Technology, 1 Castle Point, Hoboken, 07030, NJ, USA.
[3*]Quantum Computing Inc., 5 Marine View Plaza, Hoboken, 07030, NJ, USA.

*Corresponding author(s). E-mail(s): lmcevoy@stevens.edu;
Contributing authors: dtafone@stevens.edu; ysua@stevens.edu; yhuang5@stevens.edu;

1 LiDAR System Design

A further illustration of the single-photon LiDAR system that we used to image the physically masked data is shown in Figure 1.

2 Transformers

The introduction of the Transformer model in 2017 marked a significant milestone in Artificial Intelligence, propelling an AI boom with its novel encoder-decoder architecture that leverages multihead attention mechanisms. This architecture surpassed the capabilities of traditional Recurrent Neural Networks (RNNs), such as Long-short-term memory (LSTM) units and Gated Recurrent Units (GRU), by offering a more efficient and scalable approach to processing sequences. Initially, Transformers were designed for Natural Language Processing (NLP) tasks, setting a new standard for performance in this domain.

Transformers rely on a mechanism known as scaled dot product attention to assess the significance of different sections of the input relative to each other. Within this framework, three matrices are generated from the input: a query matrix Q, a key matrix K, and a value matrix V. The transformer model produces its output by

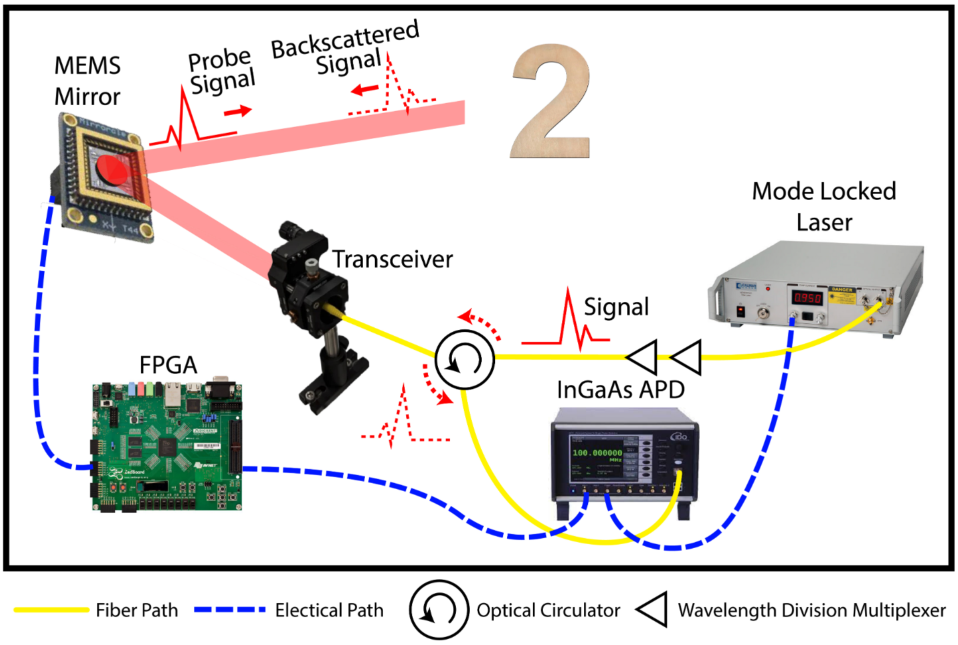

Fig. 1 System Design

calculating a weighted sum of the values, where the weights are derived from the dot product between each query and its corresponding keys, adjusted by dividing by the square root of the dimensionality of the keys, d_k.

$$\text{Attention(Q, K, V)} = \text{softmax}\left(\frac{QK^T}{\sqrt{d_k}}\right)$$

For any given pair of query and key vectors, a single score can be computed as

$$a_{i,j} = \text{softmax}\left(\frac{q_i k_j^T}{\sqrt{d_k}}\right)$$

This process, known as self-attention, focuses on identifying interactions within the dataset, as depicted in Figure 3. Advancing to Multi-Head Self Attention (MHSA), this technique enhances self-attention by processing the inputs in smaller, parallel batches across multiple subspaces. This approach significantly outperforms the sequential processing of recurrent neural networks (RNNs), where each computation depends on the output of the previous layer. In MHSA, the results of these independent attention calculations are merged as they are computed in parallel on a GPU.

$$\text{head} = \text{Attention}\left(X_q W_i^q, X_k W_i^k, X_v W_i^v\right)$$
$$\text{MultiHeadAttention}\left(X_q, X_k, X_v\right) = \left([\text{head}_1, ..., \text{head}_h]\right) W^o$$

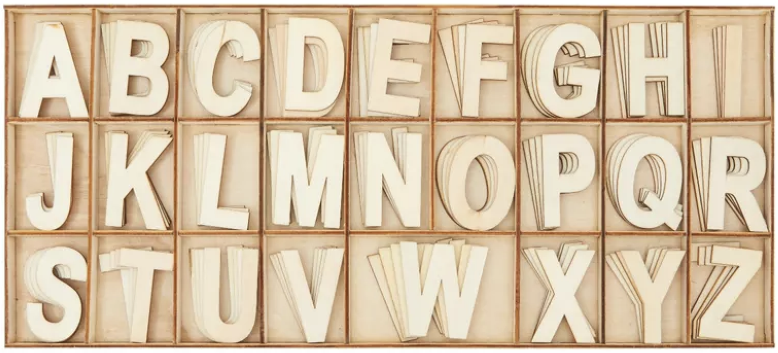

Fig. 2 Wooden Letters used to scan with single photon LiDAR system.

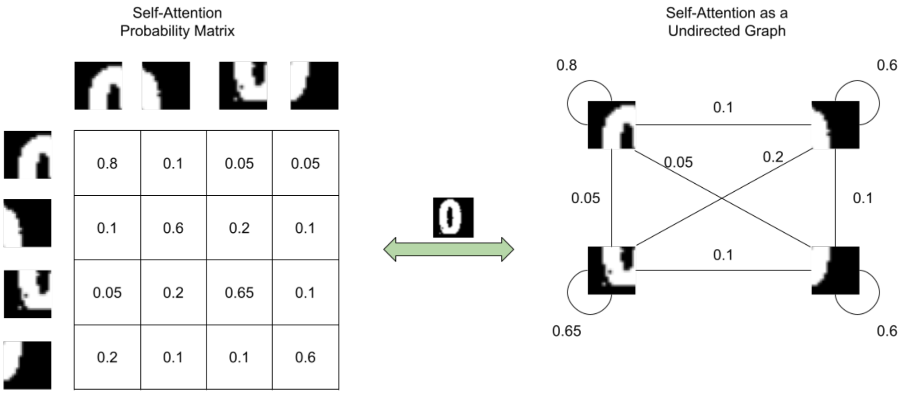

Fig. 3 Self Attention Computation

Here, W^q, W^k, and W^v denote the weight matrices for the query, key, and value, respectively, while X_q, X_k, and X_v represent the corresponding elements transformed into embedding vectors. Multiplication of these weight matrices with the embedding vectors produces the inputs for the self-attention mechanism. In the case of MHSA, the results of each self-attention calculation are combined and then multiplied by an output weight matrix W^o, which determines the relative influence of each head within the MHSA module. The distribution of influence among heads in a MHSA module typically varies, with h indicating the total number of heads.

Discussing the encoder-decoder structure, the encoder utilizes Multi-Head Self Attention (MHSA) to create an attention-focused representation of the input. This representation enables the identification and retrieval of specific information or concepts from a vast latent space. Structurally, the encoder consists of a repeated module, executed six times, featuring two key components: a MHSA layer and a fully connected

feed-forward neural network (FFNN). In an FFNN, information progresses in a singular direction—forward—without any backloops or cycles characteristic of a Recurrent Neural Network (RNN). Additionally, each sub-module is enhanced with a residual connection and layer normalization for improved performance and stability.

Turning to the decoder, its role within the transformer architecture is to extract and interpret information from the encoder's output. While the decoder's design mirrors that of the encoder, a notable distinction is the inclusion of an extra MHSA sub-module in the decoder, making two in total. The initial MHSA layer in the decoder is designed to be masked, obscuring future information embeddings. This masking ensures that the decoder cannot prematurely access information about upcoming events, aligning the model's predictions more closely with actual sequential dependencies.

Positional encoding is pivotal due to the permutation invariance of self-attention; that is, the order of data input or processing does not influence the model. To counteract this, positional encoding is essential to impart sequence awareness to the model. Without such encoding, transformers would lack the capability to discern the sequence of words in text or the order of patches in an image. These positional embeddings are designed to match the dimensionality of the input embeddings, allowing for integration with the input data.

3 Masked Autoencoder

Transformers expanded beyond NLP with the introduction of Vision Transformers (ViT) in June 2021, which applied the Transformer architecture to Computer Vision (CV) without relying on Convolutional Neural Networks (CNNs). This approach, which treats images as sequences of patches, similar to words in a sentence, opened new avenues for applying attention mechanisms to visual data.

Following ViT, Facebook AI Research (FAIR) unveiled Masked Autoencoders (MAE) in December 2021. MAEs extend the Transformer's application by masking a significant portion of the input image during training, pushing the boundaries of self-supervised learning. This method allows the model to reconstruct images from sparse inputs, demonstrating the Transformer's versatility and the power of self-attention in learning high-level data representations. MAEs utilize an asymmetric encoder-decoder architecture, where the encoder processes visible patches, and the lightweight decoder focuses on reconstructing the entire image.

4 Physics Informed Masked Autoencoder

Our PI-MAE model differs from MAE in that the scanning pattern is communicated with the patch encoder. Without this optimization, there is no way to reconstruct the physically masked images with MAE. The reason being that MAE randomly masks an unmasked input. Therefore, if we input a physically masked image into MAE, MAE will *further* mask it, causing further information loss. However, with PI-MAE, we inform the patch encoder on where we looked by providing the scanning pattern. Therefore, the Patch Encoder can intelligently decide where to mask and where to unmask, without losing information. All masked patches will be areas that we did not observe, and all unmasked patches will be areas that we did observe. Although we

observed a patch, that does not mean there is data, but this itself is informative. In the same way observing an edge gives us information on the boundaries of an object, a patch with no data informs us that we have surpassed the boundary of our scanned object.

MAE does the following. MAE gets an input image with 100% of the data present. MAE then passes this input image into its Patch Encoder. The Patch Encoder turns the input image into patches. Next, the patches are turned into embeddings, positions, and indices of patches. The masking procedure occurs in the past step, in the Patch Encoder. Here, the input image **randomly** goes from 100% to 25% (if there were a 75% masking percentage set). We will revisit the Patch Encoder for PI-MAE discussion as key optimizations in this module allow for physically masked single-photon image reconstruction without the ground truth image. With the unmasked 25% of the image, its corresponding embeddings are passed into the encoder. It is key to note that the patch encoder and the encoder are two different modules. In addition to receiving the embeddings of unmask patches, we provide the corresponding positions of these unmasked patches to Encoder.

Now that the lightweight encoder has been provided the information on unmasked patches it needs, we move on to the Decoder. We input the encoder outputs and the masked embeddings (the embeddings corresponding to masked patches) into the Decoder. This input is then decoded in the Decoder and then turned into patches via the Patch Layer. We are now left with an array of reconstructed patches, which we can reshape back into the input image dimensions.

PI-MAE is provided an input image with 25% of the data. It is important to note how this differs from MAE. PI-MAE is provided with masked data with no ground truth, unlike MAE. Furthermore, PI-MAE receives the scanning pattern that was used to acquire this 25% physically masked image. Instead of passing the input image into the Patch Encoder and having it randomly mask the input image like MAE, PI-MAE provides the scanning pattern and the input image to the Patch Encoder. The Patch Encoder in PI-MAE then intelligently uses the scanning pattern to align the mask and unmask patch locations with that of the scanning pattern. Therefore, in PI-MAE, all the locations that the scanning apparatus viewed are unmasked patches, and all the locations that the scanning apparatus did not view are masked patches. This key optimization with the Patch Encoder (from the original MAE architecture) allows for the reconstruction of physically masked images without a ground-truth image, which MAE cannot do. The rest of the operations with the Encoder and Decoder are the same as those of MAE after this patch encoder optimization in PI-MAE.

Operational Comparison

MAE Workflow: MAE starts with a fully unmasked input image, which is segmented into patches by the Patch Encoder. These patches are then randomly masked according to a set percentage, significantly reducing the data used for encoding. The encoder module receives only the embeddings of the unmasked patches, along with their spatial information, before passing them to the decoder for reconstruction.

PI-MAE Workflow: PI-MAE begins with an input image that is already 25% unmasked, reflecting the physically observed data without ground truth. The Patch

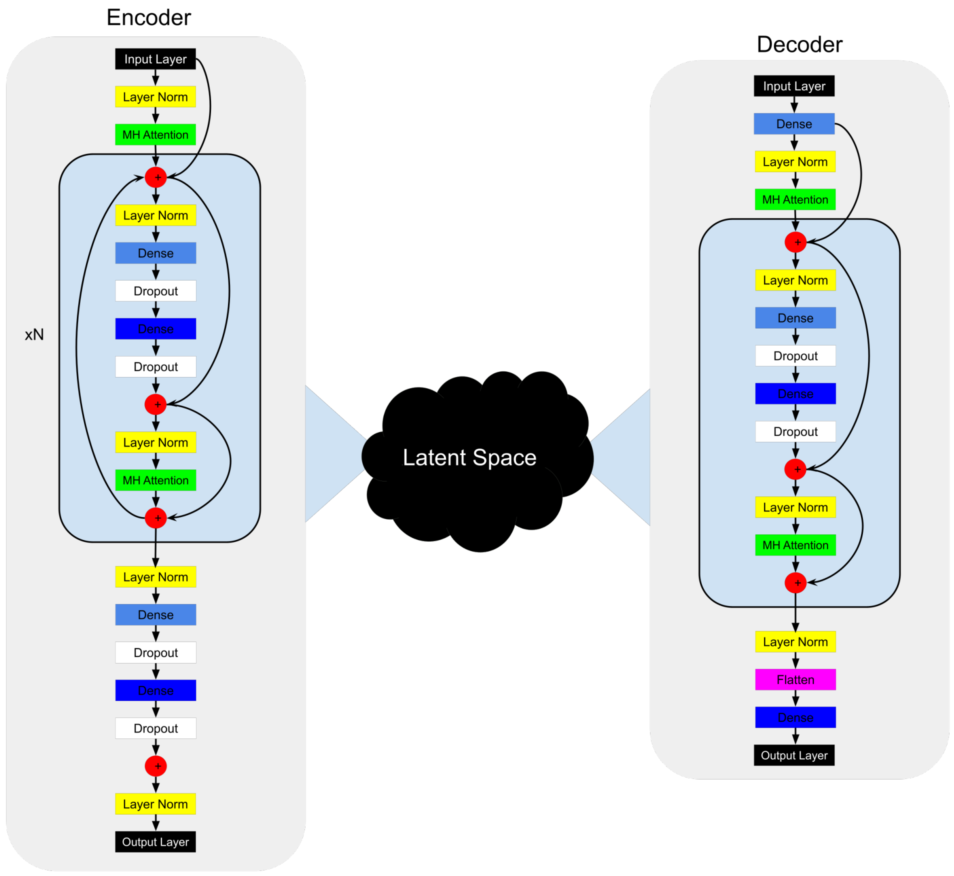

Fig. 4 PI-MAE Model Architecture

Encoder, informed by the scanning pattern, aligns masking with unobserved areas, ensuring a direct correlation between scan patterns and patch encoding. *This crucial adjustment enables PI-MAE to reconstruct images from physically masked inputs effectively, a feat unachievable by MAE.* The subsequent encoding and decoding processes mirror those of MAE, albeit optimized for physically masked inputs.

5 Benchmarking

We benchmark PI-MAE against two industry standard algorithms. The first leverages the principles of fluid dynamics and Navier-Stokes partial differential equations. This method aims to investigate edges from known to unknown regions, maintaining the continuity of edges and matching gradient vectors at the boundary. The second method utilizes the Fast Marching method to inpaint images. The algorithm considers a small neighborhood around each boundary pixel, replacing the pixel with a normalized weighted sum of known neighboring pixels. The Fast Marching Method ensures

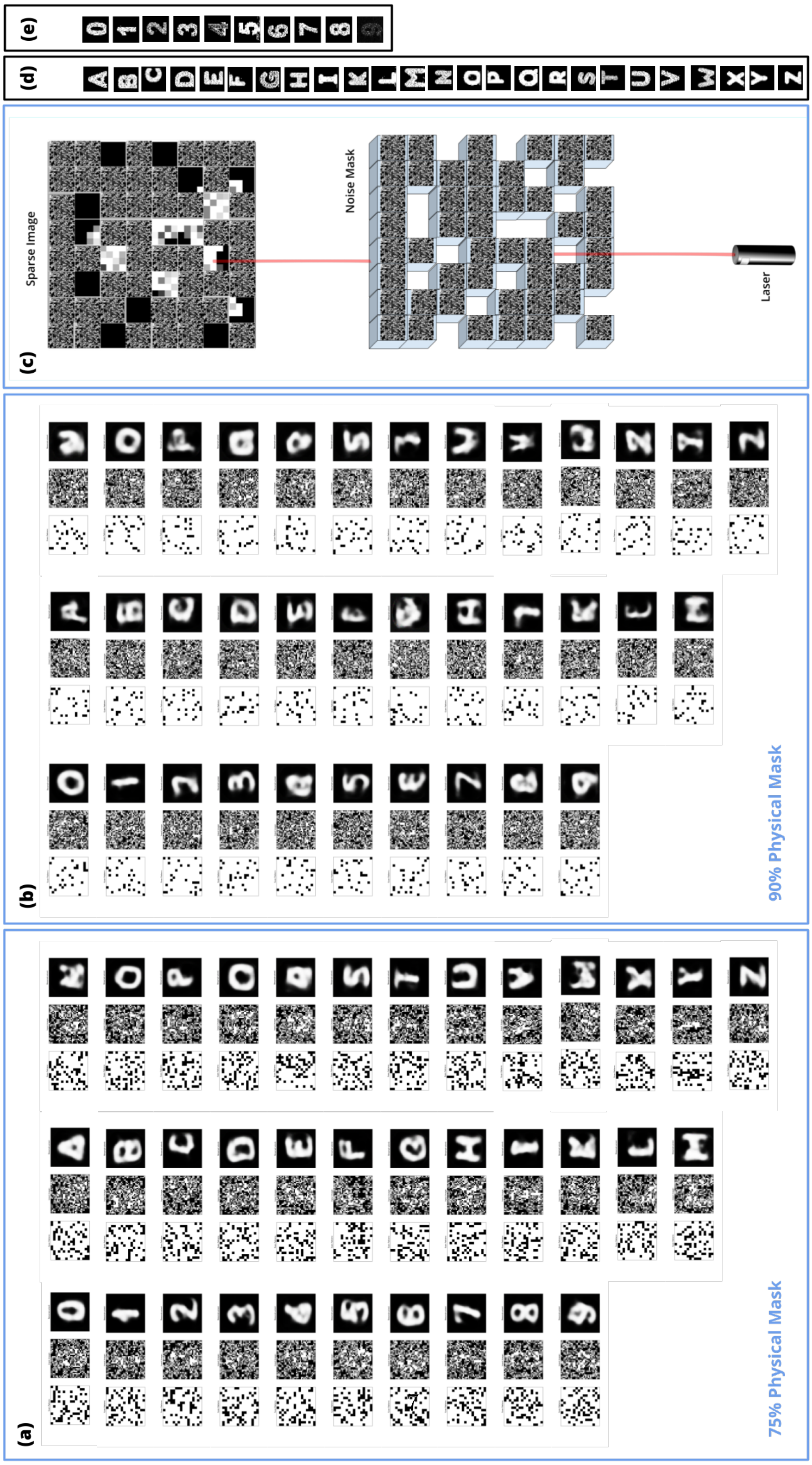

Fig. 5 Reconstructed images by PI-MAE for (a) 75% noise masking and (b) 90% noise masking. In each group of (a) and (b), the leftmost column is the scanning pattern, the middle is the sparse image collected by the LiDAR system, the rightmost column is the reconstructed images. (c) An example acquired noise masked data. (d) and (e) are the Ground-truth LiDAR scans for letters and numbers, respectively, which are what the data would have looked like if there had been no noise mask.

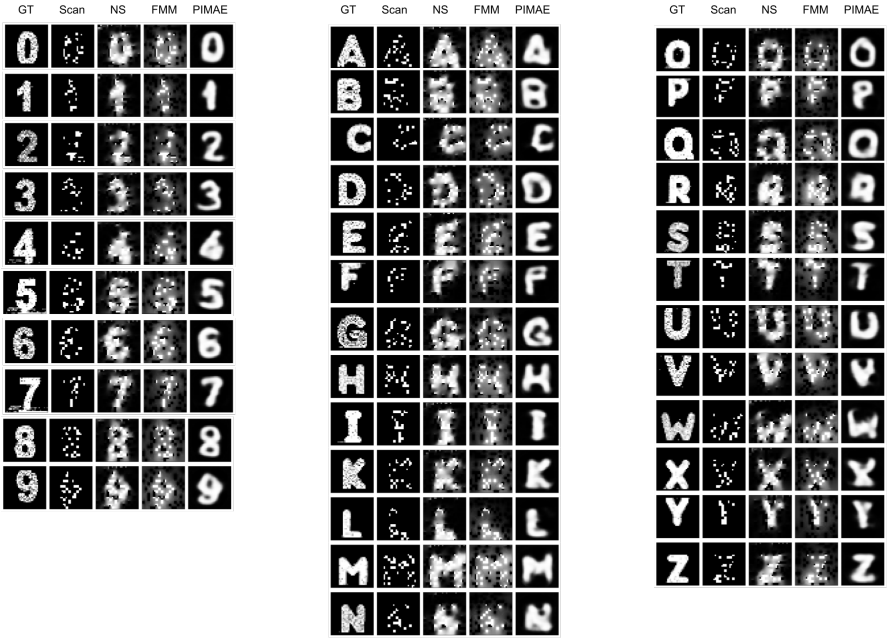

Fig. 6 Quantitative comparison for 75% physically masked scan reconstructions. Comparisons are show of Ground Truth (GT) and reconstruction methods: Naiver-Stokes (NS), Fast Marching Method (FMM), Physics-Informed Masked Autoencoder (PIMAE).

that pixels near known regions are prioritized for inpainting. Next, we performed qualitative evaluations of PI-MAE's reconstructions versus these two algorithms using three metrics: Peak Signal-to-Noise Ratio (PSNR), Structural Similarity Index Measure (SSIM), and Mean Squared Error (MSE), where PI-MAE outperformed the rivaling metrics in all three categories. Furthermore, reconstruction results between Naiver-Stokes, the Fast Marching Method and PIMAE can be seen for the 75% mask and the 90% mask in 6 and 7 respectively.

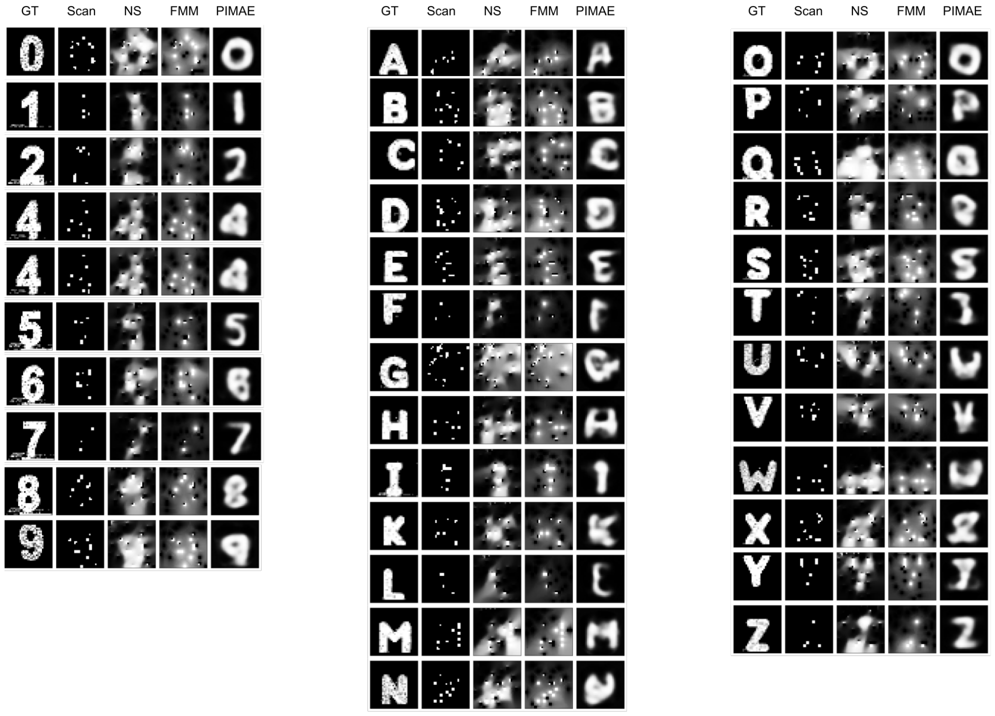

Fig. 7 Quantitative comparison for 90% physically masked scan reconstructions. Comparisons are show of Ground Truth (GT) and reconstruction methods: Naiver-Stokes (NS), Fast Marching Method (FMM), Physics-Informed Masked Autoencoder (PIMAE).
